# Real-World Outcomes of Patients with Refractory or Relapsed *FLT3*-ITD Acute Myeloid Leukemia: A Toulouse-Bordeaux DATAML Registry Study

**DOI:** 10.3390/cancers12082044

**Published:** 2020-07-24

**Authors:** Pierre-Yves Dumas, Sarah Bertoli, Emilie Bérard, Laetitia Largeaud, Audrey Bidet, Eric Delabesse, Thibaut Leguay, Harmony Leroy, Noémie Gadaud, Jean Baptiste Rieu, Jean-Philippe Vial, François Vergez, Nicolas Lechevalier, Isabelle Luquet, Emilie Klein, Audrey Sarry, Anne-Charlotte de Grande, Arnaud Pigneux, Christian Récher

**Affiliations:** 1 Service d’Hématologie Clinique et de Thérapie Cellulaire, CHU Bordeaux, F-33000 Bordeaux, France; thibaut.leguay@chu-bordeaux.fr (T.L.); harmony.leroy@chu-bordeaux.fr (H.L.); anne-charlotte.de-grande@chu-bordeaux.fr (A.-C.d.G.); arnaud.pigneux@chu-bordeaux.fr (A.P.); 2 Université de Bordeaux, 33076 Bordeaux, France; 3 Institut National de la Santé et de la Recherche Médicale, U1035, 33000 Bordeaux, France; 4 Service d’Hématologie, Institut Universitaire du Cancer de Toulouse Oncopole, Centre Hospitalier Universitaire de Toulouse, 31000 Toulouse, France; bertoli.sarah@iuct-oncopole.fr (S.B.); largeaud.laetitia@iuct-oncopole.fr (L.L.); Gadaud.Noemie@iuct-oncopole.fr (N.G.); sarry.audrey@iuct-oncopole.fr (A.S.); recher.christian@iuct-oncopole.fr (C.R.); 5 Université Toulouse III Paul Sabatier, 31000 Toulouse, France; delabesse.eric@iuct-oncopole.fr (E.D.); vergez.francois@iuct-oncopole.fr (F.V.); 6 Cancer Research Center of Toulouse, UMR1037 INSERM, ERL5294 CNRS, 31000 Toulouse, France; 7 Service d’Epidémiologie, Centre Hospitalier Universitaire de Toulouse, 31000 Toulouse, France; emilie.berard@univ-tlse3.fr; 8 INSERM-Université de Toulouse III, UMR 1027, 31000 Toulouse, France; 9 Laboratoire d’Hématologie Biologique, Institut Universitaire du Cancer de Toulouse Oncopole, Centre Hospitalier Universitaire de Toulouse, 31000 Toulouse, France; rieu.jeanbaptiste@iuct-oncopole.fr (J.B.R.); luquet.isabelle@iuct-oncopole.fr (I.L.); 10 Laboratoire d’Hématologie Biologique, CHU Bordeaux, F-33000 Bordeaux, France; audrey.bidet@chu-bordeaux.fr (A.B.); jean-philippe.vial@chu-bordeaux.fr (J.-P.V.); nicolas.lechevalier@chu-bordeaux.fr (N.L.); emilie.klein@chu-bordeaux.fr (E.K.)

**Keywords:** acute myeloid leukemia, *FLT3*-ITD mutation, primary induction failure, relapse, tyrosine kinase inhibitors

## Abstract

Two recent phase 3 trials showed that outcomes for relapsed/refractory (R/R) *FLT3*-mutated acute myeloid leukemia (AML) patients may be improved by a single-agent tyrosine kinase inhibitor (TKI) (i.e., quizartinib or gilteritinib). In the current study, we retrospectively investigated the characteristics and real-world outcomes of R/R *FLT3*-internal tandem duplication (ITD) acute myeloid leukemia (AML) patients in the Toulouse-Bordeaux DATAML registry. In the study, we included 316 patients with *FLT3*-ITD AML that received intensive chemotherapy as a first-line treatment. The rate of complete remission (CR) or CR without hematological recovery (CRi) was 75.2%, and 160 patients were R/R after a first-line TKI-free treatment (*n* = 294). Within the subgroup of R/R patients that fulfilled the main criteria of the QUANTUM-R study, 48.9% received an intensive salvage regimen; none received hypomethylating agents or low-dose cytarabine. Among the R/R *FLT3*-ITD AML patients with CR1 durations < 6 months who received intensive TKI-free treatment, the rate of CR or CRi after salvage chemotherapy was 52.8%, and these results allowed a bridge to be transplanted in 39.6% of cases. Finally, in this QUANTUM-R standard arm-matched cohort, the median overall survival (OS) was 7.0 months and 1-, 3- and 5-year OS were 30.2%, 23.7% and 21.4%, respectively. To conclude, these real-world data show that the intensity of the second-line treatment likely affects response and transplantation rates. Furthermore, the results indicate that including patients with low-intensity regimens, such as low-dose cytarabine or hypomethylating agents, in the control arm of a phase 3 trial may be counterproductive and could compromise the results of the study.

## 1. Introduction

Internal tandem duplication (ITD) in the *FLT3* gene is one of the most frequent mutations found in acute myeloid leukemia (AML) [1]. *FLT3*-ITD is associated with poor prognosis [2] and has emerged as a relevant therapeutic target [3]. *FLT3*-ITD is usually conserved at relapse, suggesting that *FLT3*-ITD AML-initiating cells are key targets for long-lasting remission [2,4]. FLT3 tyrosine kinase inhibitors (TKI), developed as ATP-competitive inhibitors, are currently the focus of new development strategies in *FLT3*-mutated AML, both as single agents at relapse and in combination with intensive chemotherapy or as first-line maintenance therapy. Type I inhibitors, including midostaurin, gilteritinib, and crenolanib, have activity against *FLT3*-ITD and tyrosine kinase domain (TKD) mutations [5]. Type II inhibitors, including sorafenib and quizartinib, do not have activity against *FLT3*-TKD mutations [5]. Although first-generation FLT3 TKI, such as midostaurin [6] and sorafenib [7], have marginal single-agent activity in an active disease, they have recently shown promising activity as maintenance therapies [8,9], notably after allogeneic hematopoietic stem cell transplantation (HSCT) [10]. Conversely, several FLT3 TKIs (quizartinib, crenolanib, and gilteritinib) have single-agent activity [11,12,13] able to provide complete or near-complete remission, providing a strong rationale to combine these agents with cytotoxic chemotherapy.

Recently, the QUANTUM-R trial, which was designed specifically for refractory or relapsed (R/R) *FLT3*-ITD AML patients, demonstrated the greater efficacy of quizartinib as a single agent versus the control arm [11]. There is no universally accepted standard approach to treat R/R AML beyond the need for enrollment into clinical trials. Commonly used therapeutic options include intermediate- or high-dose aracytine (I/HDAC)-based salvage chemotherapy, a short course of chemotherapy prior to reduced-intensity conditioning and allogeneic HSCT, low-dose aracytine (LDAC) or azacytidine (AZA), and finally, best supportive care (BSC) alone [14]. In the QUANTUM-R trial, the investigator determined which protocol-designated conventional care regimen was the most appropriate for each patient based on age, performance status, comorbidities, and/or institutional practice. In this trial, overall survival (OS) was improved in the quizartinib arm versus the control arm with a hazard ratio (HR) at 0.76 (^95%^ CI: 0.58–0.98) and a median OS at 6.2 months (^95%^ CI: 5.3–7.2) in the quizartinib arm versus 4.7 months (^95%^ CI: 4.0–5.5) in the control arm (*p* = 0.02). Composite complete remission was 48.2% in the quizartinib arm and 27.0% in the standard arm.

Therefore, the objective of the current study was to describe the characteristics, treatments, and outcomes of R/R *FLT3*-ITD AML patients treated before the era of FLT3 TKI.

## 2. Methods

The current study included patients with newly diagnosed AML according to the WHO 2016 classification [15], excluding acute promyelocytic leukemia, between 1 January 2000, and 31 December 2017. Patients were included if they were older than 18 years of age, treated by intensive chemotherapy [16], with a *FLT3*-ITD mutation and an ITD/wild type (wt) ratio > 0.03, and had received no previous treatment for AML except hydroxycarbamide. Written informed consent was obtained in accordance with the Declaration of Helsinki, allowing for the collection of clinical data in the anonymized database. Cytogenetic risk was defined in accordance with the UK Medical Research Council classification [17]. Salvage treatments were based on single-agent cytarabine (high-dose cytarabine, 3 g/m^2^/12 h, d1–4; intermediate dose-cytarabine, 1 g–1.5 g/m^2^/12 h, d1–4, or 1 g/m^2^/d, d1–5), combination of an anthracycline plus cytarabine (daunorubicin 60 mg/m^2^/d, d1–3, or idarubicin 12 mg/m^2^/d, d1–3, or amsacrine 200 mg/m^2^/d; d1–3 + cytarabine 1.5–3 g/m^2^/12 h, d1–4), or the FLAG-IDA regimen (fludarabine 30 mg/m^2^/d, d1–5 + cytarabine 2 g/m^2^/d, d1–5 + idarubicin 10 mg/m^2^/d, d1–3 + G-CSF 5 µg/kg/d, d1–5). Response to treatment, OS, event-free survival (EFS), relapse-free survival (RFS) and cumulative incidence of relapse (CIR) was defined according to the European Leukemia Net 2017 [14]. ELN 2017 considers primary induction failure as no complete remission (CR) (Bone marrow blasts < 5%; absence of circulating blasts and blasts with Auer rods; absence of extramedullary disease; absolute neutrophil count ≥ 1.0 × 109/L; platelet count ≥ 100 × 109/L) or CR without hematological recovery (CRi) (All CR criteria except for residual neutropenia < 1.0 × 109/L or thrombocytopenia < 100 × 109/L) after two courses of intensive induction treatment excluding patients with death in aplasia or death due to indeterminate cause. QUANTUM-R study included patients who were refractory to at least one cycle of a standard anthracycline-containing or mitoxantrone-containing acute myeloid leukaemia therapy. With the objective to perform the best-matched study to QUANTUM-R trial, we also applied this inclusions criterion to the current cohort, and we defined primary refractory AML as no CR or CRi after at least one course of induction chemotherapy. Relapse was defined as bone marrow blasts ≥ 5% or reappearance of blasts in the blood, or development of extramedullary disease [14].

We described the patients’ characteristics at diagnosis and at relapse using numbers and frequencies for qualitative data, and the median, interquartile range (IQR), and range (minimum–maximum) for quantitative data. For survival analyses of RFS, EFS, and OS, Kaplan-Meier survival curves were drawn and described using median, IQR, and the survival at 1, 3, and 5 years. For relapse (CIR), Cumulative Incidence Functions were drawn (since non-relapse mortality was treated as a competing event) and described using CIR at 1, 3, and 5 years. Hazard ratios (HR) and 95% confidence intervals (^95%^ CI) were assessed using a standard Cox model for RFS, EFS, and OS, and a proportional sub-distribution hazard model (an extension of the Cox model) for competing risks for CIR [18]. For the rate of CR or CRi, odds ratios (OR) and 95% confidence intervals (95% CI) were assessed using a standard logistic regression model. Multivariate analyses for newly diagnosed *FLT3*-ITD AML patients initially included all potential risk factors (age, performance status [ECOG], AML status (de novo or secondary AML), gender, white blood cells, cytogenetics risk, *NPM1* co-mutation and allogeneic hematopoietic stem cell transplantation [HSCT; only for EFS, RFS, CIR, and OS]) associated with endpoints with a *p*-value < 0.20 in univariate analyses. Stepwise regression was then used to assess variables that were significantly and independently associated with the endpoints (*p*-value < 0.05). The proportional hazard assumption was tested for each covariate of the Cox model using log-log plot method curves and was always supported. When the linear hypothesis was not supported, continuous potential confounding factors were transformed into ordered data. Interactions between variables that were significantly and independently associated with endpoints were tested in final models. None were significant. Allogeneic HSCT was evaluated as a time-dependent qualitative covariate. All reported *p*-values were two-sided, and the significance threshold was < 0.05. Statistical analyses were performed using STATA^®^ version 14.2 (STATA Corp., College Station, TX, USA).

## 3. Results

### 3.1. Patients and Treatment

Among 3290 newly diagnosed AML patients included in the DATAML registry between 2000 and 2017, 1500 did not have the information for *FLT3* mutation and 358 did not receive intensive treatment. A total of 316 patients with *FLT3*-ITD AML fulfilled the inclusion criteria (Figure S1). Their characteristics are presented in Table 1. Of these, 140 patients (44.3%) were 60 years of age or older. Extramedullary involvement and leukostasis were observed in 119 (40.9%) and 49 (15.8%) patients, respectively. The median white blood cell count (WBC) was 51.5 × 10^9^/L (IQR, 21.3–118.1) at diagnosis, and 66 (47.1%) of patients carried an *ITD*/wt ratio > 0.5. There were 279 (88.6%) cases of de novo AML. The majority of patients (295, 93.4%) had an intermediate cytogenetic risk: 238 (75.3%) had normal karyotypes, and 196 (65.1%) had a *NPM1* co-mutation.

The first induction course was idarubicin and cytarabine in 186 (58.9%) patients, with and without lomustine in 94 (29.7%) and 92 (29.1%) patients, respectively. The combination of daunorubicin and cytarabine was used in 124 (39.2%) patients, and 124 patients (42.8%) received hydroxycarbamide as a cytoreductive treatment before intensive chemotherapy. Seventy-eight patients (24.8%) were admitted to the intensive care unit either during induction therapy or in the first 3 months following the first induction course. Twenty-two patients (7%) received an FLT3 TKI associated with the first induction course: four patients (1.3%) received quizartinib in the QUANTUM-FIRST clinical trial (NCT02668653), and 18 (5.7%) received ponatinib in the PONATINIB-AML clinical trial (NCT02428543). These patients were described but excluded from efficacy and survival analyses. Among the 294 patients who received a first induction course without an FLT3 TKI, the response to chemotherapy was CR1 or CRi1 in 221 (75.2%) patients and failure in 48 (16.3%) patients. The remaining 25 (8.5%) patients died by day 30.

**Figure 1:**
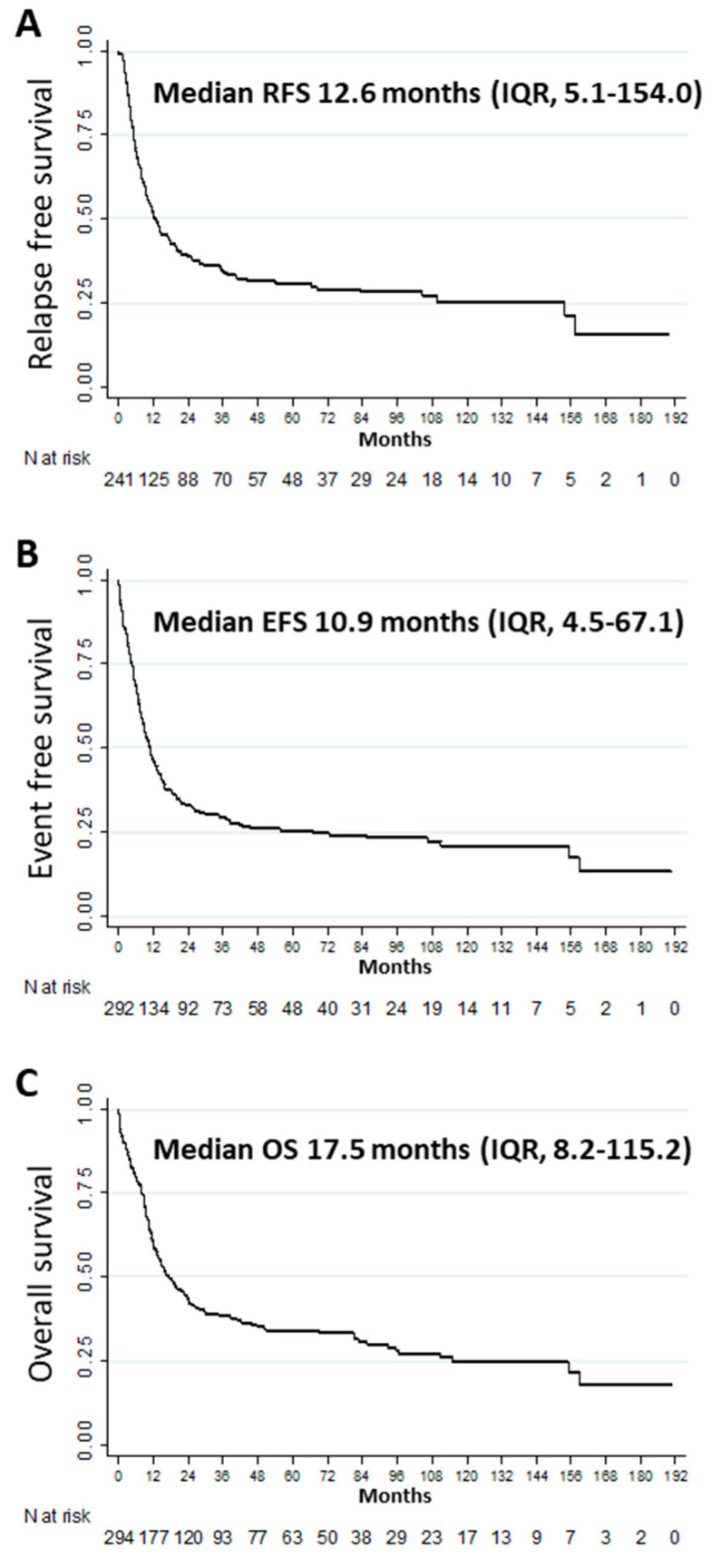
Outcomes among patients with newly diagnosed *FLT3*-ITD acute myeloid leukemia: (**A**) relapse-free survival (*n* = 241), (**B**) event-free survival (*n* = 292), (**C**) overall survival (*n* = 294).

After a median follow-up of 6 years (IQR, 3.9–9.9), 126 (57.0%) patients in CR1/CRi1 relapsed. The median RFS, EFS, and OS were 12.6 (IQR, 5.1–154), 10.9 (IQR, 4.5–67.1), and 17.5 (IQR, 8.2–115.2) months, respectively (Figure 1A–C). The CIR was 40.0% (^95%^ CI: 34.0–46.0), 53.0% (^95%^ CI: 46.0–59.0), and 57.0% (^95%^ CI: 50.0–3.0) at 1, 3 and 5 years, respectively. Other outcomes at 1, 3, and 5 years are described in Table 2. Multivariate analysis showed that age ≥ 60 years (aHR 1.70 [^95%^ CI: 1.28–2.26], *p* < 0.001), female gender (aHR 0.71 (^95%^ CI: 0.54–0.94), *p* = 0.018), and performance status ≥ 2 (aHR 1.74 (^95%^ CI: 1.26–2.39), *p* = 0.001) were significantly and independently associated with OS (Table S1). Multivariate analyses for factors associated with CR1/CRi1, RFS, CIR, and EFS are presented in the supplementary data (Tables S2, S3, S4, and S5, respectively).

### 3.2. Outcomes of R/R FLT3-ITD Acute Myeloid Leukemia Patients

Among 126 relapsing patients, 12 (9.5%) were *FLT3*wt at relapse and were therefore excluded from analyses; 46 (40.4%) relapsed within the first 6 months after CR1/CRi1, and 68 (59.6%) relapsed thereafter. Among these 114 patients, 28 (24.6%) relapsed after allogeneic HSCT. The median time to relapse was 7.2 months (IQR, 4.3–12.6). Among 46 refractory patients, 26 (56.5%) and 20 (43.5%) received one and two induction courses, respectively. The characteristics and treatment regimens of these 160 R/R *FLT3*-ITD AML patients are shown in Table 3. Thirty-nine (24.4%) patients received only BSC and not salvage therapy; 64 (40%) received I/HDAC-based salvage chemotherapy; 24 (15%) received an FLT3 TKI-based regimen; ten (6.3%) received a GO-based salvage chemotherapy; and six (3.8%) patients and one (0.6%) patient received a hypomethylating agent (AZA, decitabine) or LDAC, respectively. Sixteen patients (10%) received other types of treatment. Among the 121 patients who received a second-line treatment, 14 received quizartinib as a single agent. These patients were excluded from efficacy analyses. Three more patients were also excluded because of loss of follow-up. Among the 104 patients who received salvage treatment other than quizartinib, the response to treatment was CR or CRi in 51 (49.0%) patients and failure in 50 patients (48.1%). The three remaining patients (2.9%) died before evaluation. Among the 104 patients who received a second-line treatment, 30 (28.8%) proceeded to receive an allogeneic HSCT.

**Figure 2:**
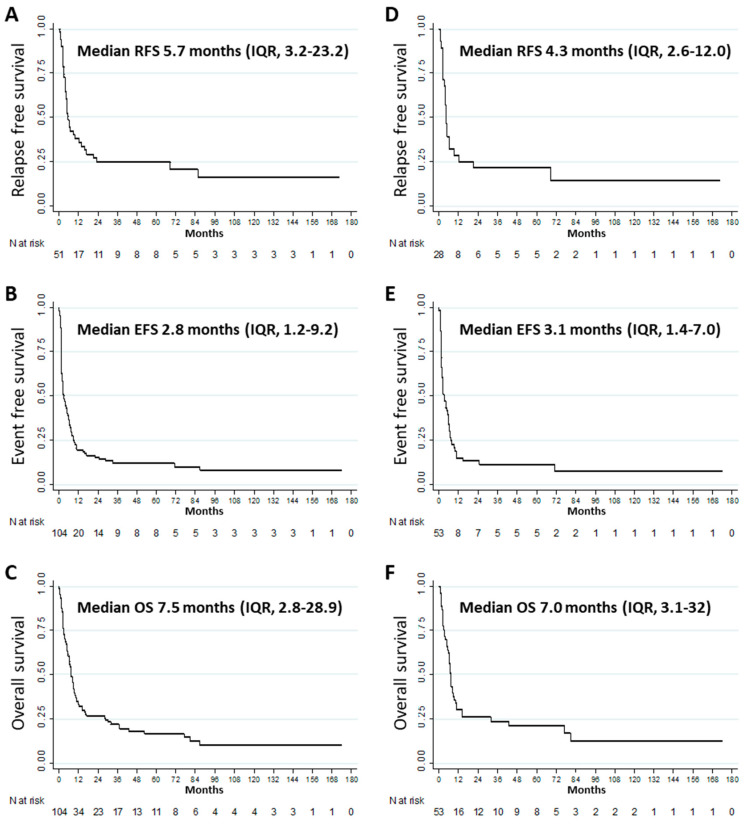
Outcomes among patients with refractory or relapsed *FLT3*-ITD acute myeloid leukemia: (**A**) relapse-free survival (*n* = 51), (**B**) event-free survival (*n* = 104), (**C**) overall survival (*n* = 104). Outcomes among patients with refractory or relapsed *FLT3*-ITD acute myeloid leukemia < 6 months: (**D**) relapse-free survival (*n* = 28), (**E**) event-free survival (*n* = 53), (**F**) overall survival (*n* = 53).

The median RFS, EFS, and OS were 5.7 (IQR, 3.2–23.2), 2.8 (IQR, 1.2–9.2), and 7.5 (IQR, 2.8–28.9) months, respectively (Figure 2A–C). Other outcomes at 1, 3, and 5 years are described in Table 2. Multivariate analyses for factors significantly and independently associated with the various endpoints were not sufficiently powered to draw any conclusions [19]. Nevertheless, in univariate analysis, duration of CR1 or CRi1 (< 6 months vs. ≥ 6 months) was significantly associated with RFS (median = 2.6 (IQR, 1.1–6.2) versus 13.6 (IQR, 4.9–85.6) months, *p* = 0.014) and was not significantly associated with EFS (*p* = 0.119), OS (*p* = 0.144) and second CR or CRi (*p* = 0.928). Moreover, *NPM1* co-mutation was not significantly associated with RFS (*p* = 0.374), EFS (*p* = 0.231), OS (*p* = 0.140) and second CR or CRi (*p* = 0.433).

### 3.3. Subgroup Analysis of Refractory or Relapsed Patients with Duration of First Remission < 6 Months

Since the QUANTUM-R study included relapsed patients with a CR1 duration < 6 months, we excluded 68 patients within our cohort who experienced relapse more than 6 months after CR1. The median time to relapse was 3.6 (IQR, 2.5–4.9) months in this subgroup. The characteristics and treatment regimens of these 92 R/R *FLT3*-ITD AML patients are shown in Table 4. Twenty-six (28.3%) patients received only BSC and not salvage treatment, 40 (43.5%) patients received I/HDAC-based salvage chemotherapy, 14 (15.2%) received an FLT3 inhibitor-based regimen, five (5.4%) received GO-based salvage chemotherapy, and none received a hypomethylating agent or LDAC. Seven patients (7.6%) received other treatments. Among the 66 patients who received salvage treatments, 11 received quizartinib as a single agent. These patients were excluded from efficacy analyses. Two other patients were excluded because of loss of follow-up. Among the remaining 53 patients who received salvage treatment other than quizartinib, the response to treatment was CR or CRi in 28 (52.8%) patients and failure in 25 (47.2%) patients. Among the 53 patients who received a second-line treatment, 21 (39.6%) proceeded to receive an allogeneic HSCT.

The median RFS, EFS, and OS were 4.3 (IQR, 2.6–12.0), 3.1 (IQR, 1.4–7.0), and 7.0 (IQR, 3.1–32) months, respectively (Figure 2D–F). Other outcomes at 1, 3, and 5 years are described in Table 2. Multivariate analyses for factors significantly and independently associated with the various endpoints were not sufficiently powered to draw any conclusions [19].

## 4. Discussion

AML patients unable to achieve CR or CRi after standard induction chemotherapy, or whose disease relapses after achieving remission, are likely to die from their disease. Ganzel et al. showed that in a large cohort of AML patients included in nine trials from 1984 to 2008, the median OS from relapse was about 6 months, with a five-year OS of about 10% [20]. Apart from time to relapse or refractory status, *FLT3*-ITD mutation and a high-risk cytogenetic profile strongly impact prognosis [21]. It is imperative to identify strategies to decrease the relapse rate and improve the efficacy of treatment at relapse for this specific population. In the QUANTUM-R trial for patients with R/R *FLT3*-ITD AML, single-agent quizartinib was found to significantly improve the median OS from 4.7 months to 6.2 months compared with the investigators choice of treatment. However, there are few directly comparable data for the control arm of the QUANTUM-R trial, except for the UK NCRI AML15, AML16, and AML17 trials, which included a total of 264 *FLT3*-ITD AML patients with a median age of 51 years. In this study, 17% of patients experienced subsequent remission, with a median OS of 2.8 months and a 1-year OS rate of 13%. Among patients with post-relapse treatment information, 65% were treated intensively, 8% were treated non-intensively, and 20% were treated with BSC; the other 7% received experimental therapies. When analyses were restricted to those treated intensively, the median OS was 4.3 months and the one-year OS rate was 17%. Among 215 patients who had not relapsed post-transplantation, 25% received an allogeneic HSCT, with a 1-year OS rate of 42%.

In the current study, we examined the characteristics and outcomes of R/R *FLT3*-ITD AML patients in a routine setting in order to evaluate the efficacy of standard treatments that appeared to have limited efficacy in the QUANTUM-R study. The main patient characteristics were similar between the salvage chemotherapy group of QUANTUM-R and the current cohort. However, 4.4% of patients received a low-intensity regimen as a salvage treatment in our study, whereas about 25% of patients were allocated to the LDAC group in the QUANTUM-R study. This factor is likely a major contributor to the discrepancy in response rates between studies. Following intensive salvage chemotherapy, the rate of CR or CRi was 49.0% in our cohort compared to 27.0% in the standard arm of the QUANTUM-R study. However, at least for the response to salvage therapy, these real-world data indicate that the intensity of the second-line treatment is likely important, and that mixing patients with low- and high-intensity regimens in a control arm may lead to confusing results. Moreover, the transplantation rate in our study (28.8%) was higher than in the standard arm of the QUANTUM-R trial (11%). These results were not affected by analyzing only relapsed patients with a CR1 duration < 6 months, even though the transplantation rate was higher (39.6%). Thus, the higher response and transplantation rates may explain the better outcomes observed in the current cohort. We recently reported the same trends in the formal comparison of real-world data *versus* ADMIRAL study, a phase III randomized trial that compared gilteritinib versus standard of care in R/R *FLT3* mutated AML [22]. There were two major differences between QUANTUM-R and ADMIRAL study criteria which are important to keep in mind to interpret data. Contrary to QUANTUM-R, ADMIRAL study could include patients with *FLT3*-TKD mutations and the duration of CR1 was not limited to 6 months. Both criteria may have contributed to select patients with more favorable characteristics in the ADMIRAL study since *FLT3*-TKD mutations do not share aggressiveness of *FLT3*-ITD and duration of CR1 is a well-established criteria associated with response and outcome to salvage therapy (Table S6).

One potential source of bias in the current study is *FLT3* status, which was known in 54% of all patients added to the DATAML registry between 2000 and 2017. However, the *FLT3* status is known in 67% of patients selected for intensive chemotherapy and 81% of patients with intermediate cytogenetic risk selected for intensive chemotherapy. Moreover, *FLT3* ITD/wild type ratio has not been studied in multivariate analyses because it was available only for 44.3% of patients. It should also be noted that the present study reflects routine practice in two French centers that cover a large proportion of the French population. Finally, there may also be measurement bias in the comparison of the remission rates based on real-world data versus an experimental arm of a prospective trial, although this measurement bias would not apply to OS or to the rate of allogeneic HSCT following salvage treatment.

The adverse effects of salvage treatments were not specifically addressed in this study, since it is well-established that high-intensity regimens (such as FLAG-IDA or MEC regimens or equivalents) are disadvantageous in terms of their use of healthcare resources, duration of stay in the hospital, transfusion support, the occurrence of infection, and quality of life. Given the dismal results of these second-line therapeutic strategies, the treatment of R/R *FLT3*-ITD AML patients remains a major challenge. FLT3 inhibitors such as quizartinib, gilteritinib, and crenolanib are interesting options for inducing responses with fewer adverse events and may support significantly improved quality of life for patients [23]. However, although FLT3 inhibitors have now become the standard treatment for R/R *FLT3*-ITD AML, the response rates and long-term OS must be improved. The combination of FLT3 inhibitors with chemotherapy or other targeted therapies is currently being studied in several randomized controlled trials, and the results are expected to bring hope for this very difficult-to-treat population.

## Figures and Tables

**Table 1:** Baseline characteristics of 316 newly diagnosed intensively treated *FLT3*-internal tandem duplication (ITD) acute myeloid leukemia (AML).

Characteristics	Total *n* = 316 (100%)
Age (years) at Diagnosis	
Median (IQR)	57.4 (47.7–67.7)
Range	18.6–81.4
Gender: *n* (%)	
Male	155 (49.1)
Female	161 (50.9)
ECOG at Diagnosis: *n* (%)	
0–1	202 (72.9)
≥ 2	75 (27.1)
WBC at Diagnosis (× 10^9^/L)	
Median (IQR)	51.5 (21.3–118.1)
Range	0.4–433.0
Tumor Burden: *n* (%) Extramedullary	
Yes	119 (40.9)
No	172 (59.1)
Leukostasis	
Yes	49 (15.8)
No	262 (84.2)
LDH	
> normal	280 (92.4)
normal	23 (7.6)
Biochemistry: Med (IQR)	
Creatinine (µM/L)	80.5 (64.0–102.0)
Albumin (g/L)	36.0 (32.0–40.0)
Fibrinogen (g/L)	4.0 (2.9–5.3)
AML Status: *n* (%)	
de novo	279 (88.6)
Secondary AML ^a^	36 (11.4)
Cytogenetic Risk: *n* (%)	
Favorable	6 (1.9)
Intermediate	295 (93.4)
Normal	238 (75.3)
Different from normal	57 (18.0)
Adverse	15 (4.7)
ELN 2010 Prognosis: *n* (%)	
Favorable	6 (2.0)
Intermediate-1	230 (76.4)
Intermediate-2	50 (16.6)
Adverse	15 (5.0)
*FLT3* Ratio ITD/wt: *n* (%)	
3–25%	34 (24.3)
26–50%	40 (28.6)
> 50%	66 (47.1)
*NPM1*: *n* (%)	
Mutation	196 (65.1)
No mutation	105 (34.9)
*IDH1/2* Mutations: *n* (%)	
*IDH1* ^R132^	10 (6.7)
*IDH2* ^R140^	7 (4.7)
*IDH2* ^R172^	0 (0.0)
No mutation	132 (88.6)

ECOG, performance status; WBC, white blood cells; AML, acute myeloid leukemia; ELN, European Leukemia Net; ITD, internal tandem duplication; wt, wild type; Med, median; IQR, interquartile range. ^a^ non-de novo AML.

**Table 2:** Outcome of *FLT3*-ITD AML patients in first line or R/R situation.

Outcome	Median Months (IQR)	1 Year % (95% CI)	3 Years % (95% CI)	5 Years % (95% CI)
First-line FLT3 Inhibitor-free Intensive Chemotherapy (*n* = 294)				
RFS	12.6 (5.1–154)	51.9 (45.4–58.0)	35.0 (29.0–41.1)	30.8 (24.9–36.9)
EFS	10.9 (4.5–67.1)	45.9 (40.1–51.5)	29.7 (24.5–35.0)	25.4 (20.4–30.7)
OS	17.5 (8.2–115.2)	60.2 (54.4–65.5)	38.3 (32.6–43.9)	33.8 (28.3–39.5)
Relapsed or Refractory FLT3-ITD Acute Myeloid Leukemia Patients (*n* = 104)				
RFS	5.7 (3.2–23.2)	38.1 (24.8–51.3)	24.7 (13.5–37.5)	24.7 (13.5–37.5)
EFS	2.8 (1.2–9.2)	19.7 (12.7–27.9)	12.0 (6.5–19.3)	12.0 (6.5–19.3)
OS	7.5 (2.8–28.9)	33.2 (24.3–42.3)	22.0 (14.4–30.7)	16.6 (9.7–25.1)
Relapsed < 6 Months or Refractory FLT3-ITD Acute Myeloid Leukemia Patients (*n* = 53)				
RFS	4.3 (2.6–12.0)	28.6 (13.5–45.6)	21.4 (8.7–37.8)	21.4 (8.7–37.8)
EFS	3.1 (1.4–7.0)	15.1 (7.0–26.0)	11.3 (4.6–21.4)	11.3 (4.6–21.4)
OS	7.0 (3.1–32)	30.2 (18.5–42.7)	23.7 (13.2–36.0)	21.3 (11.3–33.5)

IQR, interquartile range; CI, confidence interval; RFS, relapse-free survival; CIR, the cumulative incidence of relapse, EFS, event-free survival; OS, overall survival, AML, acute myeloid leukemia.

**Table 3:** Characteristics of patients with R/R *FLT3*-ITD AML (*n* = 160).

Characteristics	R/R *FLT3*-ITD AML *n* = 160 (100%)
Age (years)	
Median (IQR)	58.6 (44.8–69.7)
≥ 75: *n* (%)	18 (11.6)
Sex: *n* (%)	
Male	79 (49.4)
Female	81 (50.6)
Status: *n* (%)	
Refractory	46 (28.8)
One induction course	26 (16.3)
Two induction courses	20 (12.5)
Relapse	114 (71.3)
< 6 months	46 (28.8)
≥ 6 months	68 (42.5)
Duration of CR1 or CRi1 (months)	
Median (IQR)	7.2 (4.3–12.6)
Allogeneic HSCT history: *n* (%)	
	28 (17.5)
Previous midostaurin or sorafenib: *n* (%)	
	0 (0)

IQR, interquartile range; CR, complete remission; CRi, CR with incomplete hematological recovery, AML, acute myeloid leukemia.

**Table 4:** Characteristics of patients with R/R < 6 months *FLT3*-ITD AML (*n* = 92).

Characteristics	R/R *FLT3*-ITD AML *n* = 92 (100%)
Age (years)	
Median (IQR)	58.8 (44.4–69.4)
≥ 75: *n* (%)	8 (8.7)
Sex: *n* (%)	
Male	46 (50.0)
Female	46 (50.0)
Status: *n* (%)	
Refractory	46 (50.0)
1 induction course	26 (28.3)
2 inductions courses	20 (21.7)
Relapse	46 (50.0)
Duration of CR1/CRi1 (months)	
Median (IQR)	3.6 (2.5–4.9)
Allogeneic HSCT history: *n* (%)	
	9 (9.8)
Previous midostaurin or sorafenib: *n* (%)	
	0 (0)

IQR, interquartile range; ECOG, performance status; CR, complete remission; CRi, CR with incomplete hematological recovery; WBC, white blood cells AML, acute myeloid leukemia.

## Supplementary Materials

The following are available online at https://www.mdpi.com/2072-6694/12/8/2044/s1, Figure S1: Flow chart, Table S1: Cox model for factors independently associated with overall survival among *FLT3*-ITD AML patient, Table S2: Logistic regression model for factors independently associated with CR1 and CRi1 among *FLT3*-ITD AML patients, Table S3: Cox model for factors independently associated with RFS among *FLT3*-ITD AML patients, Table S4: Cox model for factors independently associated with CIR among *FLT3*-ITD AML patients, Table S5: Cox model for factors independently associated with EFS among *FLT3*-ITD AML patients, Table S6: Real-world data related to the chemotherapy arms of QUANTUM-R (quizartinib) and ADMIRAL (gilteritinib) trials
