# Supplementary material for: Real-World Outcomes of Patients with Refractory or Relapsed FLT3-ITD Acute Myeloid Leukemia: A Toulouse-Bordeaux DATAML Registry Study
Source: Cancers (Basel). 2020 Jul 24;12(8):2044. doi: 10.3390/cancers12082044 (PMC7465142; doi:10.3390/cancers12082044)
Supplement: Supplementary file 1 [file cancers-12-02044-s001.pdf]

Supplementary Matrials

Real-World Outcomes of Patients with Refractory or Relapsed *FLT3*-ITD Acute Myeloid Leukemia: A Toulouse-Bordeaux DATAML Registry Study

Pierre-Yves Dumas, Sarah Bertoli, Emilie Bérard, Laetitia Largeaud, Audrey Bidet, Eric Delabesse, Thibaut Leguay, Harmony Leroy, Noémie Gadaud, Jean Baptiste Rieu, Jean-Philippe Vial, François Vergez, Nicolas Lechevalier, Isabelle Luquet, Emilie Klein, Audrey Sarry, Anne-Charlotte de Grande, Arnaud Pigneux and Christian Récher

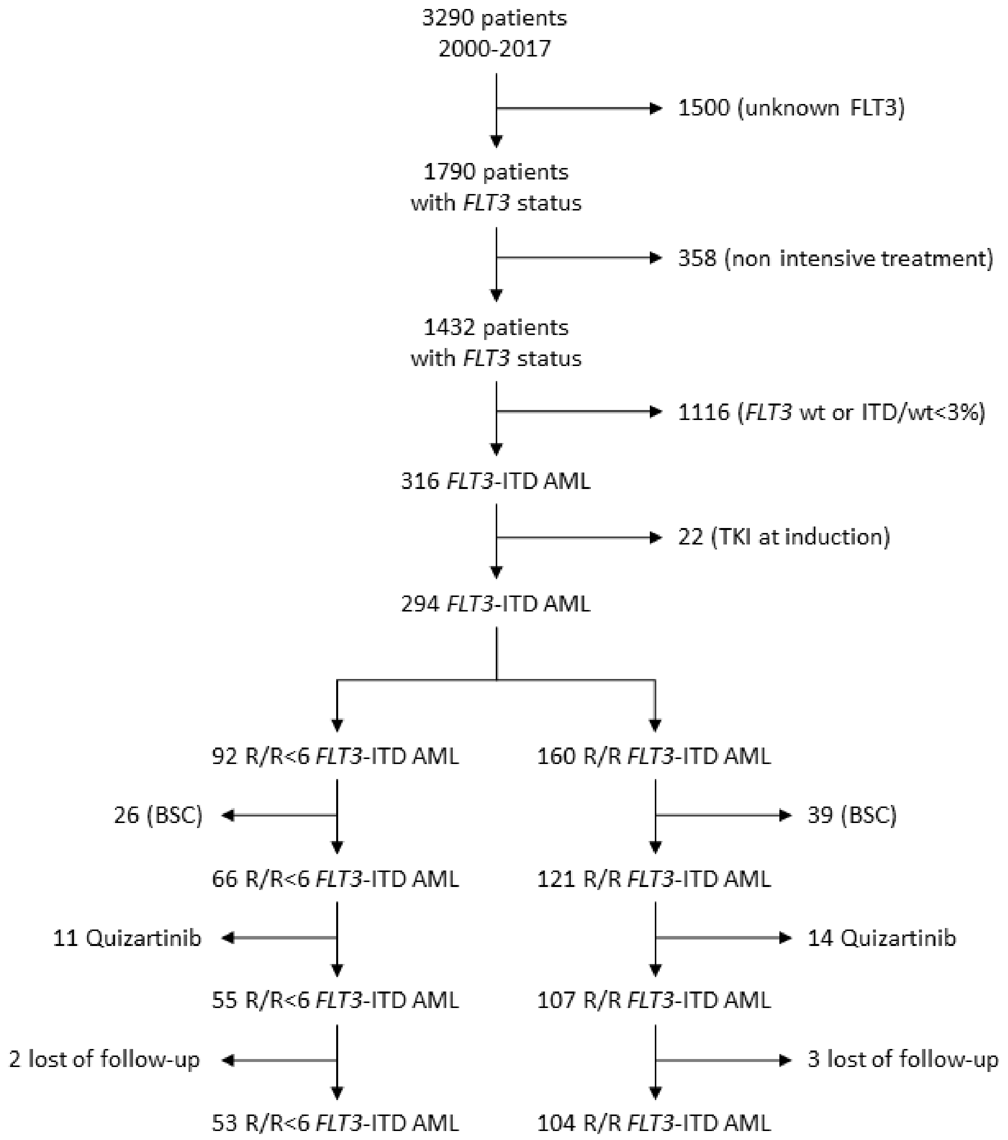

Figure 1. Flow chart.

Table S1. Cox model for factors independently associated with overall survival among *FLT3*-ITD AML patients.

Characteristics	n	Events	aHR	95% CI	P-value
Age at diagnosis					
< 60 years	167	103	1	-	-
≥ 60 years	127	98	1.70	1.28–2.26	< 0.001
Gender					
Male	146	111	1	-	-
Female	148	90	0.71	0.54–0.94	0.018
ECOG at diagnosis					
0–1	186	114	1	-	-
≥ 2	71	56	1.74	1.26–2.39	0.001

aHR, adjusted hazard ratio; CI, confidence interval; ECOG, performance status.

Table S2. Logistic regression model for factors independently associated with CR1 and CRi1 among *FLT3*-ITD AML patients.

Characteristics	n	Events	aOR	95% CI	P-value
Age subgroups					
< 60 years	167	149	1	-	-
≥ 60 years	127	94	0.36	0.18–0.71	0.003
ECOG at diagnosis					
0–1	186	167	1	-	-
≥ 2	71	53	0.45	0.21–0.96	0.040
WBC at diagnosis					
< 50 × 10^9/L	137	125	1	-	-
≥ 50 × 10^9/L	157	118	0.43	0.20–0.91	0.027
NPM1 mutation					
No	100	78	1	-	-
Yes	179	154	2.13	1.07-4.23	0.032

aOR, adjusted odds ratio; CI, confidence interval; ECOG, performance status, WBC, white blood cells.

Table S3. Cox model for factors independently associated with RFS among *FLT3*-ITD AML patients.

Characteristics	n	Events	aHR	95% CI	P-value
Age subgroups					
< 60 years	148	97	1	-	-
≥ 60 years	93	73	1.43	1.05-1.94	0.023

aHR, adjusted hazard ratio; CI, confidence interval.

Table S4. Cox model for factors independently associated with CIR among *FLT3*-ITD AML patients.

Characteristics	n	Events	aHR	95% CI	P-value
Allogeneic HSCT					
No	151	104	1	-	-
Yes	90	33	0.45	0.30–0.68	< 0.001
NPM1 mutation					
No	77	52	1	-	-
Yes	153	81	0.62	0.43-0.87	0.007

aHR, adjusted hazard ratio; CI, confidence interval; HSCT, hematopoietic stem cell transplantation (as a time-dependent covariate).

Table S5. Cox model for factors independently associated with EFS among *FLT3*-ITD AML patients.

Characteristics	N	Events	aOR	95% CI	P-value
Age subgroups					
< 60 years	166	115	1	-	-
≥ 60 years	126	106	1.65	1.26–2.15	< 0.001
ECOG at diagnosis					
0–1	184	130	1	-	-
≥ 2	71	59	1.53	1.12–2.09	0.007

aHR, adjusted hazard ratio; CI, confidence interval; ECOG, performance status.

Table S6. Real-world data related to the chemotherapy arms of QUANTUM-R (quizartinib) and ADMIRAL (gilteritinib) trials.

Characteristics	QUANTUM-R Results SOC vs. Quizartinib	Real world data *FLT3*-ITD	ADMIRAL Results SOC vs. Gilteritinib	Real world data *FLT3*-mutated [a]
Baselines characteristics of the whole cohort according to inclusion criteria of studies				
Median age (years)	56.0	58.8	62.0	58.0
FLT3-ITD mutation (%)	100	100	92.0	92.7
FLT3-TKD mutation (%)	None	None	8.0	27.7
CR/CRi (%)	27.0 vs. 48.0	52.8	15.3 vs. 34	50
Median OS (months)	4.7 vs. 6.2	7.0	5.6 vs. 9.3	8.2
Median EFS (months)	0.9 vs. 1.4	3.1	0.7 vs. 2.8	3.4
Median RFS (months)	NA	4.3	6.7 vs. 4.4	6.8
Bridge to transplant (%)	11.0 vs. 32.0	39.6	15.3 vs.25.5	34.2

[a] published in Cancers 2020, 12, 773; doi:10.3390/cancers12040773, SOC: standard of care, NA: not available.

© 2020 by the authors. Licensee MDPI, Basel, Switzerland. This article is an open access article distributed under the terms and conditions of the Creative Commons Attribution (CC BY) license (http://creativecommons.org/licenses/by/4.0/).
